# Supplementary material for: End Sequence Analysis Toolkit (ESAT) expands the extractable information from single-cell RNA-seq data
Source: Genome Res. 2016 Oct;26(10):1397–410. doi: 10.1101/gr.207902.116 (PMC5052061; doi:10.1101/gr.207902.116)
Supplement: Supplemental Material [file supp_gr.207902.116_Supp_Materials.tar.gz › ESAT-esat_v0.1/doc/html/classumms_1_1core_1_1coordinatesystem_1_1_transcriptome_space_1_1_gene_tree-members.html]

ESAT: Member List


ESAT


- Main Page
- Related Pages
- Packages
- Classes
- Files

- Class List
- Class Index
- Class Hierarchy
- Class Members

All Classes Namespaces Files Functions Variables Enumerator Pages

- umms
- core
- coordinatesystem
- TranscriptomeSpace
- GeneTree

umms.core.coordinatesystem.TranscriptomeSpace.GeneTree Member List

This is the complete list of members for umms.core.coordinatesystem.TranscriptomeSpace.GeneTree, including all inherited members.

|  |  |  |
| --- | --- | --- |
| getLength() | umms.core.coordinatesystem.TranscriptomeSpace.GeneTree |  |
| getLength(String chr) | umms.core.coordinatesystem.TranscriptomeSpace.GeneTree |  |
| getRandomAnnotation() | umms.core.coordinatesystem.TranscriptomeSpace.GeneTree |  |
| getRegion(Annotation region) | umms.core.coordinatesystem.TranscriptomeSpace.GeneTree |  |
| getRegion(String chr) | umms.core.coordinatesystem.TranscriptomeSpace.GeneTree |  |
| getRegion() | umms.core.coordinatesystem.TranscriptomeSpace.GeneTree |  |


---

Generated on Fri Aug 15 2014 15:11:10 for ESAT by  

 1.8.7
